# Using a framework to implement large-scale innovation in medical education with the intent of achieving sustainability

**DOI:** 10.1186/s12909-014-0282-1

**Published:** 2015-01-16

**Authors:** Judith N Hudson, Elizabeth A Farmer, Kathryn M Weston, John A Bushnell

**Affiliations:** Department of Rural Health, University of Newcastle, Tamworth Education Centre, 114-148 Johnston St, Tamworth, NSW 2340 Australia; Graduate School of Medicine, University of Wollongong, Wollongong, NSW Australia; Public Health, Graduate School of Medicine, University of Wollongong, Wollongong, NSW Australia

**Keywords:** Medical education, Large scale change, Longitudinal integrated clerkships, Community-based

## Abstract

**Background:**

Particularly when undertaken on a large scale, implementing innovation in higher education poses many challenges. Sustaining the innovation requires early adoption of a coherent implementation strategy. Using an example from clinical education, this article describes a process used to implement a large-scale innovation with the intent of achieving sustainability.

Desire to improve the effectiveness of undergraduate medical education has led to growing support for a longitudinal integrated clerkship (LIC) model. This involves a move away from the traditional clerkship of ‘block rotations’ with frequent changes in disciplines, to a focus upon clerkships with longer duration and opportunity for students to build sustained relationships with supervisors, mentors, colleagues and patients. A growing number of medical schools have adopted the LIC model for a small percentage of their students. At a time when increasing medical school numbers and class sizes are leading to competition for clinical supervisors it is however a daunting challenge to provide a longitudinal clerkship for an entire medical school class. This challenge is presented to illustrate the strategy used to implement sustainable large scale innovation.

**What was done:**

A strategy to implement and build a sustainable longitudinal integrated community-based clerkship experience for all students was derived from a framework arising from Roberto and Levesque’s research in business. The framework’s four core processes: chartering, learning, mobilising and realigning, provided guidance in preparing and rolling out the ‘whole of class’ innovation.

**Discussion:**

Roberto and Levesque’s framework proved useful for identifying the foundations of the implementation strategy, with special emphasis on the relationship building required to implement such an ambitious initiative. Although this was innovation in a new School it required change within the school, wider university and health community. Challenges encountered included some resistance to moving away from traditional hospital-centred education, initial student concern, resource limitations, workforce shortage and potential burnout of the innovators.

**Summary:**

Large-scale innovations in medical education may productively draw upon research from other disciplines for guidance on how to lay the foundations for successfully achieving sustainability.

## Background

Initiatives developed to address the global shortage and geographical maldistribution of medical workforce [1,2] have included increasing student numbers at existing medical schools and/or establishing new ones, many of the latter being situated in regional (non-capital city urban centres) or rural settings. Community and workplace desire for ‘practice-ready’ and ‘patient-centred’ graduates has prompted the development of new models of clinical education [3-6] that develop the skills and attributes which prepare graduates to work effectively in areas where they are most needed.

The longitudinal integrated clerkship (LIC) is one model that is gathering considerable support internationally [6,7]. It represents a move away from the more traditional curricular structure of clinical clerkship with its emphasis upon ‘block rotations’ and frequent changes in disciplines, to a focus upon the concept of long-term integrated medical student placements. A recent review of outcomes arising from LICs has revealed that they are being increasingly implemented in rural and urban settings [8,9]. In addition to positively influencing LIC students towards primary care and rural career choices [10], other reported outcomes include equivalent or sometimes better academic results, and higher-order clinical and cognitive skills than ‘block rotation’ peers, well-developed patient-centred communication skills and willingness to embrace increased responsibility with patients. Importantly, patients have described a LIC learning environment as learner- and patient-centred with a positive impact on their health care [11].

Outcomes such as these have prompted adoption of the LIC model in a growing number of medical schools with many using the model for a small percentage of their medical students before expanding LIC student numbers [12]. However, it is a daunting challenge to provide a longitudinal curriculum for an entire medical school class from the outset when increasing medical school numbers and medical class sizes are causing significant competition between Schools for scarce clinical supervisors.

This paper provides guidance on the key issues to be addressed when mounting such a large-scale educational program. It is based on the experience of implementing a whole class at the Graduate School of Medicine (GSM), University of Wollongong, Australia. This LIC example was implemented at a time of emerging global reform in clinical education, and was novel due to its scale and whole-class approach. The latter had been introduced by the Northern Ontario School of Medicine a year prior to the GSM on a smaller scale. Reports in the literature include descriptions of various LIC models and their outcomes, but there is little reported discussion of the challenges of establishing a whole school community-based approach. This manuscript describes the barriers, constraints and opportunities when a regional context provided the focus to promote innovation [13].

The GSM four-year graduate-entry medical program was established in 2007 aiming to produce competent, patient-centered graduates with a vocation to serve in regional, rural and remote communities. Its innovative curriculum includes a longitudinal integrated clinical clerkship for a full academic year in the third phase of the course [11,14]. All students live in their allocated community, learning and working in all its health services including primary care, hospitals and extended services. In a typical week, a student spends two days in their host general practice and one day in the hospital emergency medicine department, where they assess undifferentiated patients under the supervision of their preceptor(s). The remaining days comprise an academic day with case-based learning, simulation and/or interprofessional learning activities, and another hospital day. In the latter, the student takes part in a range of learning experiences including ward-based care, surgical assisting and obstetric care.

This LIC model places community-based health education, workplace-based learning and continuity of care [6] at the core of curriculum processes. In doing so, it challenges the traditional practice of two years of predominantly short-term discipline-based clinical education in tertiary hospitals. By creating new and extended community settings for undergraduate clinical education, the LIC model parallels changes within health services that reduce patient time in acute care settings, and uses the educational potential of the increasing availability of patients being treated in ambulatory care, primary care and community settings.

A significant challenge for the School therefore was to work effectively in these new environments to not only create an innovative curriculum model but also to secure its future by using a framework to foster sustainability. This was a major undertaking. Although the School was new, the experience of undergraduate medical education for most of staff and partners in hospitals and primary care was predominantly the traditional curriculum model of short-term clinical placements in tertiary teaching hospitals that they themselves had experienced as students. For several academics and/or clinicians used to teaching and learning in more traditional models, the innovation represented a change in approach and thus generated concern and resistance to departing from established models of clinical education.

To provide the LIC component of the course for all students, the School created new community-based ‘Teaching Microsystems’ [15,16] based in ten regional, rural and remote educational hubs in New South Wales (NSW). The curriculum and hubs have now been in operation for five student cohorts (N = 379) since 2009.

**Figure 1:**
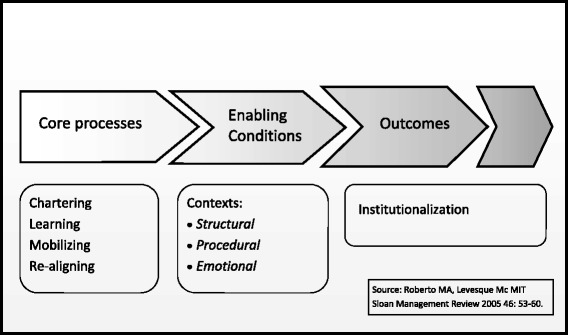
**The Change Management Framework.**

This article articulates our learning from the development and early delivery phase of this substantial change in philosophy and delivery of undergraduate clinical education. Roberto and Levesque’s model [17] (Figure 1), originally described for business, was used a guiding framework for achieving sustainability when implementing the new educational model. The framework’s four core processes and key steps in each informed the approach to rolling out the ‘whole of class’ longitudinal community-based medical education program.

## What was done

### Chartering

The senior staff of the medical school defined the purpose and scope of the initiative, as well as selecting and defining how people would work with one another on the program. It required careful delineation of our needs, creating and articulating a model for describing the intended relationships with new sites. Following visits to each location by the Dean and Associate Dean Community-based Health Education (CBHE) to promote the model, the School determined the staffing required for the various sites. Each location had a different capacity for student numbers, as well as differences in varying existing supervisor resources and experience with undergraduate medical education.

### Defining and communicating the vision

The first key step was to define the vision for the longitudinal clerkship and communicate this effectively to multiple distributed stakeholders. As the School’s overall vision was to attract and retain future rural doctors, and produce competent, patient-centred clinicians, this part of the medical course was seen as a highly visible and tangible expression of the vision within the overall curriculum model.

Communication strategies included engagement and consultation with a wide range of stakeholders; multiple site visits to communities and their practitioners and services; strategic planning workshops; and personal contacts with community leaders and organisations including local government. Through a multi-faceted approach, the School promulgated its vision and ideas for change to many potential partners. As undergraduate community-based teaching relied primarily on private practitioners with limited government funding for teaching activities, it was necessary to be clear at a very early stage about the intended benefits of belonging to the initiative, and the practicalities of how the education model would function.

### Attracting early adopters

Early in the project the School needed to seek out and attract the possible clinical teams that might have the interest to build and sustain teaching hubs, and recruit to the School the necessary staff to develop and support the initiative. In a climate of competition for clinical placement sites, and because the School sought only regional, rural and remote sites, the choice of suitable locations was limited. Inevitably, they were widely dispersed. Clinicians or practices already engaged with vocational training were sought, as well as those seeking a new model of engagement with medical schools, given the lack of ‘rural return’ from short-term student placements. Articulation of the outcomes already reported from longitudinal clerkships was motivating for practices [6,12,18-20]. The long-term preceptor-student partnership offered a significant chance to help form and develop an individual student. The active role for students was predicted to be more cost-effective than multiple short-term students [18], and to foster a high level of student competence [19,20]. Preceptors were attracted by the fact that regional and rural patients had expressed willingness for active involvement in ambulatory teaching [21], and that continuity of student-patient relationships had been reported to be conducive to developing patient-centred graduates [5].

### Defining the scope and inclusive ways of working

This initiative was distributed around NSW, a large state in Australia. Connection between the sites and ‘base’ campus was enabled by e-learning, videoconferencing and a fundamental focus on relationship building. Success required engagement of a diverse group of people: patients and the wider community; local government; health services; hospital and community health professionals; and other education providers. As the need for improving the future medical workforce in rural areas was well recognised, and communities reported limited return of short-term students to rural practice, they were very receptive to a new approach to addressing the issue. Since the University was funded explicitly to build rural medical workforce, the School could capitalize on this shared goal, establishing relationships underpinned by a philosophy of valuing partnership. Stakeholders from the new rural ‘outposts’ of the University, sharing the University’s mission of building future rural workforce became ‘shareholders’ in the initiative.

Considerable effort was invested in relationship building [22,23]. Strategies included frequent visits to each community by the senior academic and executive staff and LIC management team, and early appointment of local academic and administrative staff. Visits to each community (at least once each semester) included dinners where evidence on the outcomes of similar programs was presented, and professional development activities on clinical supervision and formative and summative assessment of students, were delivered. Each practice was visited to meet practice administrative as well as clinical staff over a working lunch. While professional relationships with potential preceptors were key to initiating the program, the commitment to establishing informal relationships with newly appointed local academic and administrative staff proved critical for maintaining the enthusiasm for the initiative in the early years.

As the project proceeded, evaluation feedback from supervising clinicians revealed their commitment to their profession, ‘handing on’ to the next generation and helping their community to attract doctors in the future [14]. Overtly recognising and valuing the preceptors for the values they held, helped build the ongoing relationship between the community and institution.

Students in the first longitudinal clerkship class were uncertain how a year-long clerkship would proceed as there were no senior peers with experience of the model. They received multiple briefings to answer questions and foster a shared commitment to the mission held and articulated by core LIC staff and involved communities. The prior rural experience (living, schooling and/or placement in rural area) of many students, as well as a presentation from a graduate of a similar program helped to build excitement about this phase of the course. An allocation system allowed students to prioritize their preferred practice and region, and over 90% received their first or second choice. Student allocation six months prior to the placement allowed time for initial relationship building, connection and planning for relocation.

## Learning

This second core process helped the team see what was needed for ‘roll-out’, including fundamental redefinition of the relationship between a University and its partners in the ‘field’. The University team had to listen to local partners and stakeholders to understand how the shared vision could be implemented using the personnel and resources in each locality.

### Testing and developing the final model

By drawing on the literature and consulting with others who had implemented smaller scale versions of the initiative in a similar context [7,19], it was possible to test and refine the model, adapting general principles to local resources and demands. For instance, a small town with no other medical students had different demands compared to a town already teaching students from other institutions and undertaking various models of clinical education. Some hubs needed to modify various features to implement the innovation in their setting, and it was not useful to tell them how it ‘had to be done’. Rather, user feedback was respected in the interest of fostering engagement, local ownership and partnership and building local solutions to local issues. Community consultative committees in each region helped ensure local interests were included in the final model.

### Proposed funding of the model

A critical part of the learning process in the ‘testing stage’ prior to roll-out was defining the funding that would support the initiative. Like all other medical schools in Australia, the University received a substantial Rural Clinical Training and Support (RCTS) grant from the Australian Government Department of Health. The RCTS program is designed to increase the rural medical workforce by enlisting Australian medical schools to deliver rural medical training, to recruit rural medical students, promote and encourage rural medical careers [24]. This funding supported the core University LIC team comprising a senior academic (program director), and two senior professional staff members, with responsibility for the development of the educational program, professional staff and infrastructure in each hub, and part-time rural hub academic coordinators and professional support staff. However, community-based preceptors were private general medical practitioners earning income on a fee-for-service basis. In addition to their altruistic motives for engagement, they required clear articulation of the incentives offered to host a longitudinal student each year.

Worley and Kitto’s hypothetical model [18] of the financial impact of a student on a rural general practice over time proved useful for preceptors to reflect on the likely impact of long- rather than short-term students. The model derived from data involving seven general practitioners supervising long-term students, challenged the view that medical students are always a financial burden on rural general practices, and was helpful for preceptor recruitment. Recruitment was also facilitated by the Aus$50,000 infrastructure grant that participating practices received for a commitment of a minimum of five years to the program. Every practice signed an individual University-practice placement agreement for receipt of this RCTS practice infrastructure grant. Funding was used to build or modify existing practice infrastructure so each LIC student had access to their own consulting room. Finally, a separate national Government funding stream provided a practice incentive payment (PIP) for teaching (Aus$100 per 3 hour consulting session). This went a small way in acknowledging the time and contribution that GPs make to student learning.

Our early evaluation data [25] have supported the hypothetical model, suggesting that students can be cost-neutral or have a small positive financial impact on the practice within a few months. This has been supported by preceptor perceptions, with most (66%) perceiving the longitudinal placement as financially neutral or favourable. Nineteen per cent of preceptors reported a negative financial impact, some attributing this to reduced patient throughput, inadequacy of the government teaching subsidy and/or time spent on assessment preparation. Other supervisors were unconcerned about costs, perceiving that minor financial loss was outweighed by personal satisfaction. Data such as this are useful for ongoing preceptor commitment.

### Defining how diverse locations will work with each other

It was important to promote personal connections between staff in all regions, as well as with the University. This was facilitated by a ‘curriculum conference’ prior to roll-out, and once or twice-yearly after commencement. These 2-day conferences brought all rural staff in the hubs back to the University. The vision and existing evidence base for LICs, and the planned curriculum were shared. While each hub had its own discrete health care micro- and meso-systems [16] and local challenges, the collective working together in small groups generated new ideas, built a team, and generated improved strategies to deal with real or imagined barriers.

## Mobilising

Mobilising entailed the use of symbolism, compelling stories, vision and sharing of data to engage institutional leaders, staff, students and community partners to build commitment to the project and energise participants.

### Storytelling

Medical practitioners have a tradition of storytelling to share and ‘debrief’ on clinical cases with colleagues and students, and reflect on outcomes. As a result, storytelling was used to engage ‘hearts as well as minds’ [17] to build commitment to the project. Examples included LIC staff and preceptors sharing stories of rural practice, and how limited rural-return had resulted from short-term student rotations. Resources that proved valuable were students and/or preceptors with prior LIC experience to share stories with target communities of practice. These proved to be compelling to garner uncommitted potential participants.

### Symbolic action

**Figure 2:**
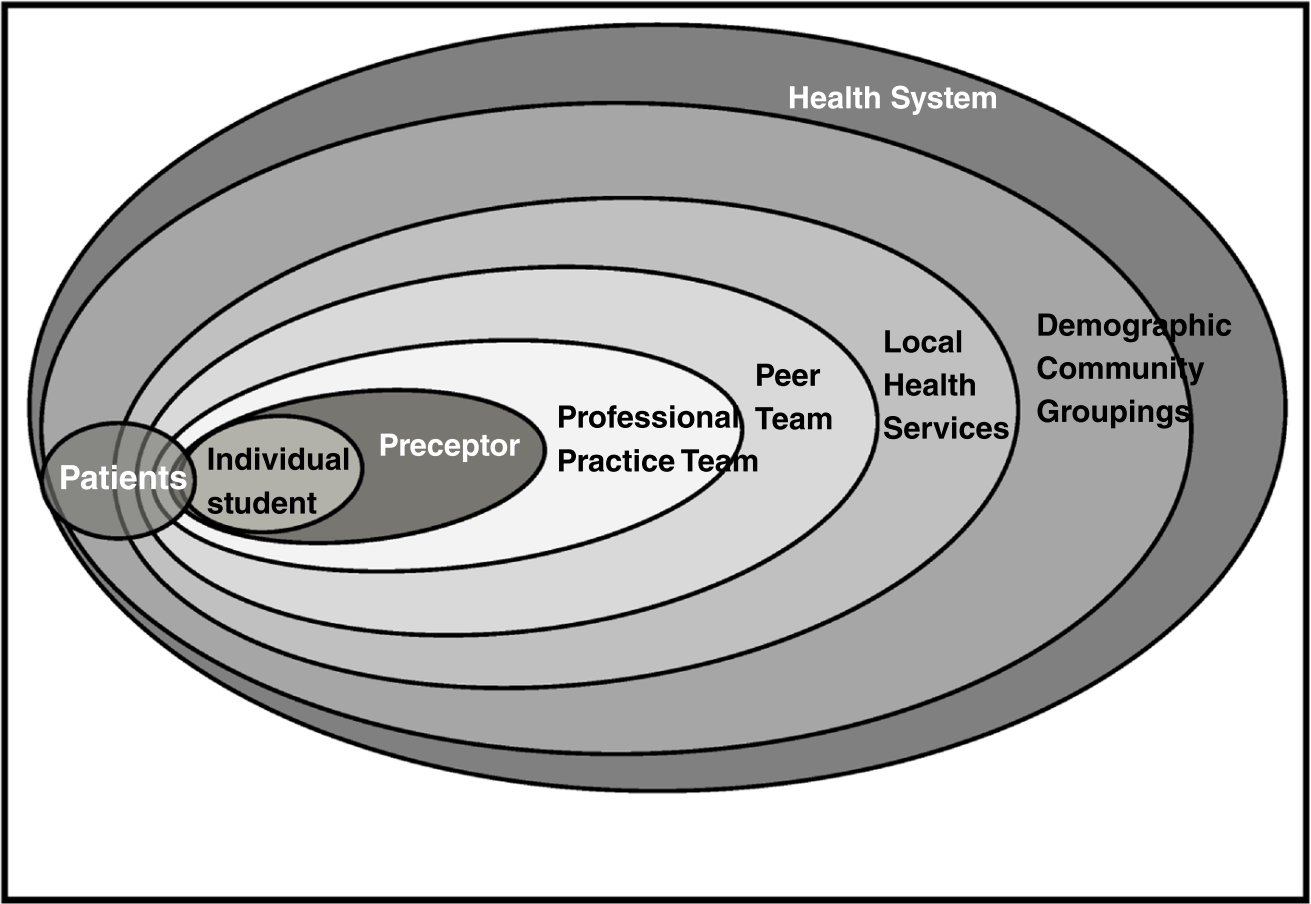
**Relationships in the longitudinal integrated community-based clerkships.**

The diagram illustrated in Figure 2 helped to symbolise the central, inclusive role for the student in the patient-student-preceptor triad and how this discrete teaching micro-environment would enable highly personalised and individualised patient-centred learning. It also highlights the range of shareholders in the initiative, from whom the student would learn during the year: other practice staff, peers in the same learning hub for academic and pastoral support; various groups in the community and regional health service; and the wider health service (macrosystem) [16]. This multi-layered ‘egg’ was supported by the institution (‘egg cup’) and the concept helped to inform the strategies for delivering a functional curriculum with local resources.

## Realigning

The final step, realigning, comprised a series of activities aimed at shaping the organisational context of the medical school, clearly defining new roles, and their reporting relationships as well as robust approaches to monitoring, and measurement, with rapid response mechanisms in case of difficulty. This was up-front work that the team had to undertake in order to build commitment to the University, ensure components of the LIC initiative became part of the School’s core processes and procedures, and make sure that University policy and procedures would be delivered at all sites.

### Creating connections

One of the key challenges was to ensure that the students, preceptors and associated community-based teaching sites and staff were well connected to the School. This required planning for flexible delivery of the curriculum including electronic delivery of core material using multiple methods. As access to the curriculum was via the web, planning included establishing good internet access for all relevant staff and students, and staff training in the development of interactive online learning modules. Another challenge involved harnessing the energy of early adopters. In addition to personal visits, relationships were consolidated by videoconferencing and personal phone contact with local academic and administrative staff. Several of the locally-based clinicians subsequently became the academic leaders of regional hubs. While they were able to advise how local resources could contribute to curriculum delivery, additional support was important to guide them through University processes.

Connections were facilitated by new rural infrastructure grants. The LIC team embraced a heavy workload to equitably distribute the Commonwealth government RCTS funding provided for this purpose. As mentioned earlier this infrastructure funding was designated for building consulting rooms, student accommodation and providing educational technology. Individual legal contracts and placement agreements, while burdensome, clearly documented the expected benefits and responsibilities associated with acceptance of funding and engagement with the initiative.

### Clarity of processes for monitoring and evaluation

All stakeholders were asked to contribute to, and accept evaluation of, the various processes. This included informal evaluation for continuous quality improvement and formal evaluation as part of being accountable to the funding body, students, the University and the public. The latter was already set up for all phases of the course, overseen by the GSM quality assurance and evaluation committee. A framework for LIC research was also developed. Engaging new staff in LIC evaluation and research helped to engage support and foster engagement.

Existing and new preceptors have recently re-engaged or joined the program after the first five years. The “academies of learning” developed through academic institution-healthcare service partnerships have provided an environment conducive to scholarship and learning, not only for medical students but for their preceptors too [26]. While it will be several years before graduates finish their postgraduate training and settle in practice in various locations, promising data are emerging about the rural career intentions of our LIC graduates [27].

The University invested significant resources to implement an electronic reflective Clinical Log for all students [28]. In addition to the educational aims for the Log, it was introduced to monitor student experience across the regions.

### Faculty development

Extensive faculty development, conducted in each site, focused particularly on teaching and learning, and on assessment in the workplace. The program of faculty development sought out the educational needs in each region and tailored educational workshops to locally identified needs. However, it also provided a forum for clarifying the process through which the University conducted and evaluated its educational mission.

### Enabling conditions

This article has described the processes that allowed regional participants to take ownership of the initiative, accepting the underpinning principles, and expressing it in their region. A number of structural, procedural and emotional enablers were also important for sustain the process as follows:

- Continued high level support of the LIC model by University leadership
- Acknowledging the concerns of those wanting to reinstitute or maintain the ‘old way’ of doing clinical education, but seeking proactive ways to overcome obstacles to change that were posed
- Committing full-time staff resources to manage the workload arising from deploying government-funded teaching infrastructure grants
- Ensuring adequate levels of administrative support were available to enable clinical supervisors to focus on what they could best provide
- Designing relevant curriculum structures and vehicles for distributed learning
- Improving and enhancing educational technology and striving for a culture of developing creative online resources
- Creating appropriate student housing options, enabled for e-learning
- Providing practical and continuing development of all academic teams
- Planning for succession at all levels
- Embracing new ideas or better ways of doing things as learning about the initiative progressed
- Ongoing articulation of the vision, purpose and excitement
- Disseminating results and celebrating early successes
- Rapid response and practical support when difficulties arose

## Discussion

Sustainability is the most difficult aspect of any change process [17]. Roberto and Levesque emphasise the need to attend to the solid and targeted foundation work that underpins later successful initiatives rather than succumbing to the following well-known scenario described in their article…*the CEO announces a bold initiative, designed to dramatically lift a company’s performance, which calls for sweeping changes in the company’s processes, systems and culture, unfolding with great fanfare and large resource investments*…*following staff burn-out and early failure, managers look back and wonder what went wrong* ([17], p.53). They argue that four critical processes pave the way for successful institutionalisation of a strategic change initiative. The important elements rely on an understanding of the mix of task-related, emotional and behavioural factors and they highlight how this diverges from conventional wisdom about programmatic innovation or change.

In 2001, Oswald and colleagues reported successful outcomes from a small scale initiative in the UK with 13 students undertaking a long-term community attachment integrated with hospital-based education [29]. As discussed above, an increasing number of medical schools have used this model of longitudinal integrated community-based education on a small scale. This paper reports on the considerable groundwork required to scale-up and offer such an experience to all students in a new medical school, addressing the following obstacles to implementation along the way: resistance to moving away from traditional tertiary hospital-centred education (the prior experience of most staff in the new school); initial student uncertainty about a new model of clinical education; resource limitations; workforce shortage with reliance on preceptors working in private practice in most sites; and potential burnout of the innovators.

To implement and deliver the large-scale LIC project, and endeavour to make the innovation ‘really stick’, the School set in motion a series of processes right from the start, which we have described using the Roberto and Levesque framework. The School created and shaped the organisational structure and demonstrated that our processes were fair and legitimate for all stakeholders. The LIC implementation team employed a range of open, personal and consultative approaches to engage people’s emotions, and where possible overcome resistance to change to help ensure that this initiative became more than just the latest fad. These extensive foundations, with a special emphasis on relationship building, were necessary to deliver the desired outcomes of community engagement, ensuring that students could attain the necessary graduate outcomes, and promoting potential rural, regional or remote vocation within the limits of sustainable costs and logistics. The programme is now in its sixth year and hopefully the strong foundations on which it was built will sustain it in to the future.

The partnership between the Australian Government Department of Health and the University to address the maldistribution of medical workforce in Australia has been crucial to the implementation of the program. Costs and sources of funding are important for sustainability. For this model of clinical education, additional infrastructure is required when medical education goes to new locations (as opposed to being delivered in developed tertiary teaching hospitals). Department of Health support, in addition to University funding, indicates the high level leadership that is required to address challenges in medical workforce. For many years, the Australian government has allocated funds to initiatives that address the rural health workforce shortage. The recent doubling of the PIP-teaching payment and further infrastructure funding for enhanced general practice engagement in teaching are positive steps towards sustaining programs such as community-based LICs in Australia. It is important to understand the difficulties, constraints and opportunities that medical schools face when implementing innovation (or change) in their own context [13]. Sharing the implementation process in our regional context aims to inform educators, clinicians or policy makers in other settings of how an implementation framework can assist in delivering large-scale innovation.

## Summary

The capacity of new educational initiatives to promote desired social goals is constrained by the need for sustainability. The LIC model used as an example in this article can only help to prepare medical workforce with the skills and attributes to work effectively in areas where they are most needed if the initiative is sustainable. At the outset of a bold educational innovation, considering deliberate ways to not only deliver but also build for sustainability may optimise the likelihood of maintaining the innovation.
